# Chemical-functional characterization of *Ascophyllum nodosum* and *Phymatolithon calcareum* and dietary supplementation in post-weaning pigs

**DOI:** 10.3389/fvets.2024.1431091

**Published:** 2024-12-12

**Authors:** Sara Frazzini, Serena Reggi, Matteo Dell’Anno, Anna Paola Fifi, Elena Scaglia, Irene Ferri, Luciana Rossi

**Affiliations:** ^1^Department of Veterinary Medicine and Animal Science (DIVAS), University of Milan, Lodi, Italy; ^2^Biotecnologie B.T. Srl, Todi, Italy; ^3^Department of Civil, Environmental, Architectural Engineering and Mathematics (DICATAM), University of Brescia, Brescia, Italy

**Keywords:** *Ascophyllum nodosum*, *Lithothamnium calcareum*, seaweeds, bioactive compounds, antioxidant, antimicrobial, functional ingredients, post-weaning pigs

## Abstract

**Introduction:**

As the livestock industry grapples with the need for sustainable land, maintaining production systems, and reducing antimicrobial resistance, the application of functional nutrition emerges as a potential solution.

**Aim:**

In line with the One Health principles, this study aims to evaluate functional properties of *Ascophyllum nodosum* and *Phymatolithon calcareum,* and assess the effects of their dietary supplementation on piglets’ health.

**Materials and methods:**

A chemical-functional characterization was conducted before and after *in vitro* digestion. Total Polyphenols Content (TPC) and Total Flavonoid Content (TFC) were determined through colorimetric assays, while antioxidant activity was determined using ABTS assay, and the microdilution method was used to evaluate the antimicrobial capacity. For the *in vivo* trial twenty-four post-weaning pigs (28 ± 2 days, 6.89 ± 0.820 Kg) were enrolled in two homogeneous groups (*n* = 12/group): control group (CTRL) fed a commercial diet, and treated group (ALGAE) fed commercial diet with the addition of 1.5% of *A. nodosum* and 0.5% of *P. calcareum* for 27 days. Weekly, zootechnical performances were assessed monitoring the body weight and the individual feed intake. Fecal samples were collected to evaluate the abundance of total, lactic acid and coliform bacteria through plate counting. Serum were obtained at day 0 and day 27 to assess the antioxidant barrier.

**Results and discussion:**

The chemical characterization discloses that the minerals’ level remains below the maximum thresholds defined for their use in piglets nutrition. TPC was 330.42 ± 21.372 mg TAE/g of the sample and 11.45 ± 0.521 mg TAE/g of the sample for *A. nodosum* and *P. calcareum*, respectively, and a similar trend was found in the TFC evaluation (213.85 ± 20.557 and 2.71 ± 0.900 mg CE/g of sample, respectively). Our results also highlighted that polyphenols and flavonoid compounds persisted after *in vitro* digestion as well as the functional properties. The administration of algae in piglets diet, although it slightly affected feed efficiency in the first period of the trial, did not affect the animal growth in terms of weight and average daily gain. Microbiological analysis of feces showed similar values between the two experimental groups over 27 days. A significantly higher serum antioxidant barrier was registered in ALGAE compared to CTRL group at day 27 (363.26 ± 16.241 vs. 230.69 ± 32.078 HClO/mL, *p* < 0.05).

**Conclusion:**

In conclusion, the supplementation with *A. nodosum* and *P. calcareum* could be considered a promising dietary strategy to enhance the oxidative barrier in weaned piglets.

## 1 Introduction

In recent years, sustainable development has become a prominent topic of discussion, fueled by the depletion of natural resources and environmental shifts caused by both population growth and the expansion of agro-industrial production (1). In this context, embracing the principles of agroecology is becoming increasingly relevant. Agroecology offers holistic and environmentally friendly approaches to agricultural practices, emphasizing the integration of ecological principles into food production systems (2). Indeed, in the livestock sector, the concept of nutritional ecology has become crucial (3). This approach emphasizes understanding the nutritional requirements of animals in their ecological context, considering factors such as diet composition, feeding behavior, and interactions with the environment. By applying principles of nutritional ecology, livestock producers can optimize feed efficiency, minimize environmental impacts, and promote animal health and welfare (4). In this scenario, another pressing concern is antimicrobial resistance (AMR), recognized by the World Health Organization (WHO) as one of the top ten public health threats worldwide, which poses a significant threat to animal health, human health, and global food security. For these reasons, given the imperative to reduce antibiotics (5) and promote their responsible use (6), exploring alternatives to antibiotics is essential especially during the critical phases of animal production. In this context, animal nutrition emerges as a pivotal factor. Beyond meeting essential nutrient requirements, functional ingredients can play a crucial role in promoting animal health and diminishing reliance on antibiotics (7). In the pig farming system, antibiotic drugs are commonly employed to manage diseases occurring during critical phases of the animal’s life, such as the weaning phase. During this period, piglets, still immature in terms of digestion and immunity, encounter various stressors including separation from the mother, environmental and social changes, hierarchical struggles for food access, transitioning from milk to starter feed, and gradual loss of passive immunity (8). These stressors activate the hypothalamic–pituitary–adrenal axis, releasing glucocorticoids that heighten susceptibility to potential pathogens (9). A frequent disorder during the weaning phase is post-weaning diarrhea (PWD), primarily attributed to certain strains of *Escherichia coli*. PWD leads to significant economic losses, elevated rates of morbidity and mortality, and a decline in productive performance (10–12). Hence, there is a clear need to reduce the occurrence of PWD enhancing animal health and the profitability of agricultural sector. Simultaneously, it’s crucial to minimize antibiotic usage to mitigate antibiotic resistance problems (13–15). With this premise, animal nutrition should aim not only to meet the nutritional requirements of animals but also to adhere to agroecological principles and contribute to the reduction of antibiotics in livestock production. In this regard, functional ingredients can be supplemented into animal feed to enhance overall health, improve performance, or address specific health concerns. Functional ingredients can include a wide range of substances such as antioxidants, natural by-products, and other bioactive compounds. Among all possible ingredients proposed, algae, emerges due to their composition, and the fact that they can be grown on non-arable land, making them valuable as functional ingredients. In addition to their nutritional qualities, they are a rich source of many biologically active compounds and one of the richest sources of natural antioxidants and antimicrobial compounds (16), this makes them ingredients deserving special attention for animal nutrition purposes. Algae are the most common organisms in aquatic environments and belong to a complex heterogeneous group in terms of ecological, taxonomic, morphological and biochemical aspects and are therefore divided into two main categories (microalgae and seaweeds) both actually used in animal nutrition (17, 18). Among these*, Ascophyllum nodosum* is a brown alga commonly studied in animal nutrition due to its content of vitamins, trace elements, lipids, carbohydrates, proteins, and iodine (19). Also, red algae are studied; among these, *Phymatolithon calcareum* (formerly *Lithothamnium calcareum*) (Rhodophyta) has been successfully used in cattle feed where, in addition to being an alternative mineral source, it can modulate the rumen pH (20, 21). A previous study conducted by our research group showed that the combination of *A. nodosum* and *P. calcareum* might exhibit a synergistic effect disclosing a high antioxidant and antimicrobial power (22).

The use of algae in animal nutrition, especially in pig farming, still needs to be explored. Existing literature reports variable and sometimes conflicting results, mainly focusing on supplementing individual algal species. Therefore, this study aimed to investigate the chemical-functional properties of *A. nodosum* and *P. calcareum in vitro*. Subsequently, these algal species were supplemented into a post-weaning pigs’ diet to evaluate their effect on zootechnical performance and health status.

## 2 Materials and methods

### 2.1 Chemical characterization

To determine the main nutritional components (ash, crude fiber, crude protein, ether extract), the “Official Methods of Analysis” according to AOAC were used (23). Briefly, dry matter (DM) was obtained by drying the samples in a forced-air oven at 65°C for 24 h (AOAC method 930.15). Ash (Ash) was obtained by placing the samples in a muffle furnace at 550°C for at least 3 h (AOAC method 942.05). Crude fiber (*CF*) was determined by the filter bag method (AOCS method Ba 6a-05) (24). Crude protein (CP) was determined by the Kjeldahl method using 6.25 as nitrogen conversion factor (AOAC method 2001.11). Ether extract (EE) was determined by ether extraction in the Soxtec system (AOAC 2003.05).

The content of micro- and macro-elements (Ag; Al; As; B; Ba; Ca; Cd; Co; Cr; Cu; Fe; K; Mg; Mn; Mo; Na; Ni; P; Pb; Sb; Se; Sr.; Th; Ti 47; Ti 48; Tl; U; V; Zn) contained in the two algae was evaluated after mineralization with inductively coupled plasma mass spectrometry (ICP-MS). Firstly, the dried algae were digested with a high-performance Microwave Digestion system Milestone ETHOS UP. Specifically, 500 mg of *A. nodosum* was diluted with 9 mL of 69% HNO_3_ plus 1 mL of H_2_O_2_ and digested by applying a one-step temperature ramp (at 190°C in 15 min and maintained for 15 min; 1800 MW). While for *P. calcareum* 250 mg of dried sample was diluted with 10 mL of 69% HNO_3_ and digested by applying a one-step temperature ramp (at 210°C in 20 min and maintained for 20 min; 1800 MW). Then, the samples were diluted to the concentration of 14.84 mg/L and 7.44 mg/L for *A. nodosum* and *P. calcareum*, respectively. Before proceeding with the ICP-MS analysis a calibration curves for each element considered were obtained using certified reference materials purchased by Agilent (Santa Clara, CA, United State). The concentration of elements was measured by ICP-MS (Agilent 7,900). The data obtained were finally analyzed with Software MassHunter Workstation for 7,900 ICP-MS, Version C.01.01 (Agilent Technologies 2015).

### 2.2 Functional characterization

#### 2.2.1 Extraction procedure

According to Gouvinhas et al. (25), solvent extraction was performed with some adaptations. Briefly, 40 mg of *A. nodosum* and *P. calcareum* dried meal were dissolved in 1.5 mL of methanol/deionized water (1:1, v/v). The mixture was then vortexed and stirred at room temperature (RT) for 30 min. Samples were centrifuged for 15 min at 10,000 rpm at 4°C. The supernatants were collected, filtered through a 0.20 μm syringe filter and stored at −20°C until the analysis.

#### 2.2.2 Evaluation of total polyphenol content (TPC)

The phenolic content of algae extracts was evaluated by the Folin–Ciocalteu method, according to Attard (26). Briefly, the assay was performed by reacting 50 μL of sample with 500 μL of 1:10 Folin–Ciocalteu reagent and 400 μL sodium carbonate (1 M). After 15 min of incubation at RT in the dark absorbance was read at 630 nm. The Total Phenolic Content (TPC) was expressed as tannic acid equivalents equivalents (mg TAE/100 g). Each sample and standard were run in triplicate.

#### 2.2.3 Evaluation of total flavonoid content (TFC)

The flavonoid content of algae extracts was evaluated according to Herald (27). Briefly, 250 μL of extract, 1 mL of distilled water, and 75 μL of NaNO_2_ (50 g/L) were combined and mixed. Next, 150 μL of AlCl_3_ (100 g/L) was added to the solution. After 6 min 500 μL of NaOH (1 mol/L) and 500 μL of distilled water were added. The solution was centrifuged at 3220 g for 5 min at RT, the supernatant was collected, and absorbance was measured at 510 nm. Total flavonoid concentration was expressed as mg catechin equivalent (CE) g-1 sample. Each sample and standard were run in triplicate.

#### 2.2.4 Evaluation of ABTS radical scavenging activity

The scavenging activity of algae extracts was evaluated through ABTS assay, as previously performed in our previous study (28). Briefly, 10 μL of the sample was added to 1 mL of ABTS^•+^ working solution. The absorbance was recorded at 734 nm after 6 min of incubation, performing all the determinations in triplicate. ABTS radical scavenging activity was expressed as the percentage of the inhibition of radical scavenging activity (PI%).

#### 2.2.5 Growth inhibition assay

Evaluation of the antimicrobial activity of *A. nodosum* and *P. calcareum* was carried out by a growth inhibition assay based on liquid culture with F4^+^ *Escherichia coli*. In detail, a culture of F4^+^ *E. coli* prepared in Luria-Bertani (LB) medium and allowed to grow overnight was used as the inoculum for the experiments. The growth inhibition assay was performed as follows: algal extracts were diluted in LB liquid medium to obtain a dilution of 1:8 (v/v). Then 100 μL of the diluted extract was placed in a 96-well microtiter plate into which 30 μL of the *E. coli* inoculum was added. Positive controls were prepared by adding 30 μL of the *E. coli* inoculum to the methanol/distilled water (1:1, v/v) solution to assess bacterial growth without external influences. Negative controls, on the other hand, were prepared by adding 30 μL of LB without the *E. coli* inoculum. Then all samples were incubated at 37°C in a shaking incubator for six hours. The growth of *E. coli* was assessed every hour for six hours by measuring the absorbance with a microplate reading spectrophotometer at an optical density (OD) of 620 nm. The measured optical density was converted to log_10_ of the number of cells/mL, considering 1 OD = 1 × 10^9^ cells/mL. All assays were performed in technical quadruplicates and with three biological triplicates that were intended to verify the replicability of the experiment using the same procedures, which included repeating the experiment from sample extraction and repeating the assay on different days (29).

### 2.3 *In vitro* digestion

*In vitro* digestion was performed according to the COST INFOGEST method, simulating the three main stages of digestion (oral, gastric, and intestinal) using salivary, gastric, and intestinal fluids made according to the protocol (Supplementary Table S1) (30). Briefly, 2 g of algae powder was mixed with 2 mL simulated saliva fluid stock solution, and 200 μL of *α*-amylase (Sigma–Aldrich A3176–500KU) solution of 1,500 U/mL was added. Then, pH was adjusted to 7 with 1 mol/L NaOH and incubated in a water bath at 37°C for 2 min with constant shaking. In the gastric phase, the oral bolus was mixed with 3.2 mL of simulated gastric fluid stock solution followed by 200 μL pepsin (Sigma–Aldrich P7000) solution of 25,000 U/mL. Then, pH was adjusted to 3 with 1 mol/L HCl, followed by an incubation during 2 h under the same conditions as in the oral phase. In the final phase, gastric chyme was mixed with 3.4 mL of simulated intestinal fluid stock solution, 2 mL of pancreatin (Sigma–Aldrich P1625) solution of 800 U/mL, and 1 mL of bile (Sigma–Aldrich B8631) and NaOH (to adjust the pH 7). NaOH (1 mol/L) was used to set the pH back to 7. A final incubation was carried out at 37°C for 2 h. Following *in vitro* digestion, the resulting undigested fraction (UF) was filtered through pre-weighed Whatman no. 54 filter paper in a porcelain funnel and dried overnight at 65°C. This dried UF was used to calculate the *in vitro* digestibility (IVD) according to the following formula (31):


$$IVD\;\left(\%\right)=\frac{\left(\mathrm{sample}\;\left(DM\right)-\mathrm{sample}\;UF\;\left(DM\right)\right)}{\mathrm{sample}\;\left(DM\right)}*100$$

### 2.4 Functional characterization after *in vitro* digestion

#### 2.4.1 Determination of total phenolic and flavonoid contents after *in vitro* digestion

At the end of each digestive phase, 2 mL aliquots of the digested fraction were taken and stored at −80°C to stop the enzymatic activity. Subsequently, they were used to evaluate the TPC and TFC during *in vitro* digestion following the procedure described in Sections 2.2.2 and 2.2.3 above.

#### 2.4.2 Determination of antioxidant and growth inhibitory activity after *in vitro* digestion

The assessment of antioxidant activity following *in vitro* digestion phases was performed by evaluating the ABTS radical scavenging activity according to Hsu et al. (32), as described in Section 2.2.4. On the other hand, the determination of the growth inhibitory activity against F4^+^ *E. coli* was determined as described in Section 2.2.5.

### 2.5 Animals, housing, experimental design, and treatment

The experimental trial was approved by the Animal Welfare Organization of University of Milan and the Italian Ministry of Health (n° 884/2021-PR) and performed following European regulations (33). The trial was held at the swine sector of the Experimental Zootechnical Education Center of the University of Milan, Faculty of Veterinary Medicine, based in Lodi. Twenty-four piglets (Landrace X Large White) weaned at 28 ± 2 days were identified by individual ear tags and randomly divided into two experimental groups (CTRL and ALGAE) homogeneous by sex (50% male and 50% female) and weight (6.89 ± 0.820 kg). Homogeneous macro- and microclimatic conditions were maintained throughout the experimental phase with passive forced ventilation, and the temperature was kept around 26°C with a relative humidity of 65%. Animals were housed, for 27 days, and after six days of an adaptation period during which the animals were fed the same basal diet, piglets were assigned to two experimental groups and were fed *ad libitum*: the control group (CTRL: 12 piglets, 12 pens, 7.81 ± 0.786 kg) was fed with the basal diet; the ALGAE group (ALGAE: 12 piglets, 12 pens, 7.75 ± 1.157 kg) fed with the basal diet supplemented with the addition of 1.5% of *A. nodosum* plus 0.5% of *P. calcareum*. All the diets were isonitrogenous and isoenergetic (Table 1) balanced using Plurimix System®software (Fabermatica, Cremona, Italy) in line with nutritional requirements for post-weaned piglets (NCR, 2012) and were provided by Ferraroni S.p.A. (Cremona, Italy). Considering the small inclusion percentage, to ensure a homogeneous dispersion of seaweeds meal, *A. nodosum* and *P. calcareum* were premixed with 5 kg of basal feed and then mixed for 20 min with the basal diet. Experimental diets were analyzed in duplicate for principal nutrient content: dry matter (DM), crude protein (CP), ether extract (EE), crude fiber (*CF*), and ash concentrations, according to “Official Methods of Analysis” (23) as described above. Moreover, the starch content was determined through the Total Starch Assay Kit purchased by Megazyme (Ireland) following the manufacture instruction.

**Table 1:** Composition of experimental diets of control (CTRL) and treatment (ALGAE) group.

Ingredients, % as fed basis	CTRL	ALGAE
Barley, meal	24.84	24.34
Wheat, meal	23.74	23.27
Soybean, meal	7.00	6.86
Corn, flakes	6.61	6.48
Corn, meal	6.55	6.42
Wheat, flakes	6.31	6.18
Soy protein concentrate	5.43	5.32
Dextrose monohydrate	2.99	2.93
Sweet milk whey	2.97	2.91
Soybean protein concentrate	2.97	2.91
Soy oil	1.98	1.94
Beet pulp	1.93	1.89
Coconut oil	1.07	1.05
Plasma, meal	1.00	0.98
Acidifiers mix^1^	0.79	0.77
L-Lysine	0.60	0.59
Herring, meal	0.59	0.58
Dicalcium phosphate	0.51	0.50
Vitamins and minerals premix^2^	0.50	0.49
Benzoic acid	0.50	0.49
DL-Methionine	0.22	0.22
L-Threonine	0.22	0.22
Tri- and Di-butyrate	0.20	0.20
Sodium chloride	0.15	0.15
L-Valine (96.5%)	0.11	0.11
Calcium carbonate	0.08	0.08
Flavor	0.05	0.05
Enzymatic mix^3^	0.05	0.05
L-Tryptophan	0.04	0.04
*Ascophyllum nodosum*	-	1.50
*Lithothamnium calcareum*	-	0.50
Calculated chemical composition		
Crude protein (%)	17.50	17.50
Ether extract (%)	4.80	4.80
Crude fiber (%)	2.90	2.90
Ashes (%)	4.30	4.30

^1^Benzoic acid, Citric acid, Folic acid. ^2^ Additives per Kg: Vitamins, pro-vitamins and substances with similar effect. Vitamin A (Retinyl acetate) 15000.00 UI, Vitamin D3 (cholecalciferol) 2000.00 UI, Vitamin B1 (Thiamine mononitrate) 2.00 mg, Vitamin B12 (Cyanocobalamin) 0.03 mg, Vitamin B2 (Riboflavin) 5.00 mg, Vitamin B6 (pyridoxine hydrochloride) 3.50 mg, Vitamin C (sodium calcium ascorbyl phosphate) 150.00 mg, Vitamin E (all-rac-alpha-tocopheryl acetate) 120.00 mg, Vitamin K3 (menadione nicotinamide bisulphite) 2.00 mg, Betaine hydrochloride 1000.00 mg, Biotin 0.20 mg, Choline chloride 300.00 mg, Niacinamide 30.00 mg, Butyl hydroxytoluene (BHT) 0.30 mg, Propyl gallate 0.15 mg, Sepiolite 576.50 mg, Bentonite 4900.00 mg, Calcium D-pantothenate 15.00 mg, Copper (Copper Oxide-I) 100.00 mg, Iodine (Calcium iodate anhydrous) 1.00 mg, Iron (Iron-II sulphate monohydrate) 120.00 mg, Manganese (Manganese-II oxide) 50.00 mg, Selenium (Sodium selenite) 0.35 mg, Zinc (Zinc oxide) 100.00 mg, ^3^ 6-phytate, Endo1,3(4)-beta-glucanated, Endo-1,4-beta-xylanation.

### 2.6 Animal performance, diarrhoea occurrence, and biological sample collection

Body weight (BW) was recorded individually at day 0 (T0), day 7 (T1), day 13 (T2), day 21 (T3), and day 24 (T4). Feed intake was recorded weekly by measuring the feed refused for each pen, considering the pen as the experimental unit. Other performance parameters: average daily gain (ADG), average daily feed intake (ADFI), and feed conversion ratio (FCR), were calculated according to the following formulas:


$$ADG\;\left(g/\mathrm{day}\right)=\frac{\mathrm{Final}\;\mathrm{weight}-\mathrm{Initial}\;\mathrm{weight}}{\mathrm{Number}\;\mathrm{of}\;\mathrm{days}}$$

$$\mathrm{ADFI}\;\left(g/\mathrm{day}\right)=\frac{\mathrm{Cumulative}\;\mathrm{feed}\;\mathrm{intake}}{\mathrm{Number}\;\mathrm{of}\;\mathrm{days}}$$

$$FCR=\frac{\mathrm{Feed}\;\mathrm{intake}}{\mathrm{Average}\;\mathrm{daily}\;\mathrm{gain}}$$

**Figure 1:**
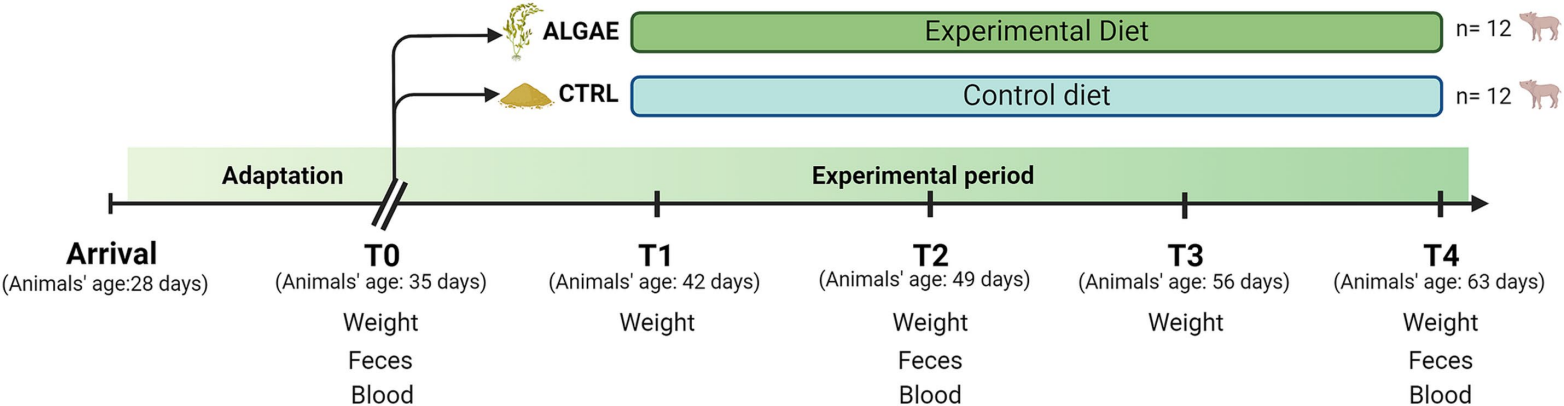
Experimental design from arrival date (28 days of animal age) to T4 (63 days of animal age) divided according to the diet administered.

Fecal samples for microbiological analysis were collected from each piglet on day 0 (T0), day 7 (T1), day 13 (T2), day 21 (T3), and day 24 (T4) (Figure 1). Diarrhea occurrence was recorded daily by evaluating the fecal consistency, which was given a fecal score: a four-level scale (0 = dried consistency, 1 = soft consistency, 2 = mild diarrhea, 3 = severe diarrhea) according to Dell’Anno et al. (34). The fecal color was evaluated through a three-level color scale: 1 = yellowish, 2 = greenish, and 3 = brown; considering ≥2 as a normal score (35). Blood samples were obtained from the jugular vein at T0, T2, and T4 through vacuum tubes without anticoagulants for oxidative barrier analysis.

### 2.7 Microbiological evaluation of fecal samples

Fecal samples were analyzed for the total countable bacteria, lactic acid bacteria, and coliform bacteria through three different types of culture media: Plate Count Agar (PCA), De Man, Rogosa, and Sharpe Agar (MRS), and Violet Red Bile Agar (VRBA), respectively. Briefly, one gram of fecal sample was diluted 1:10 with sterile 0.9% NaCl solution and homogenized by vortexing. Samples were then serially diluted tenfold, and microorganisms were enumerated by plate counting after (i) 36 h at 30°C for total bacteria and Lactic acid bacteria, (ii) 18–24 h 35°C under microaerophilic conditions for coliform bacteria (36). The lactic acid/coliform bacteria ratio was calculated using MRS and VRBA agar plate counting data. The results were expressed as log_10_ colony-forming units per gram of fresh feces (log_10_ CFU/g).

### 2.8 Protein intake and apparent protein digestibility

The following formula was used to calculate protein intake according to Augenstein et al. (37):


$$\begin{array}{l} \mathrm{Daily}\;\mathrm{protein}\;\mathrm{intake}\;\left(g/\mathrm{day}\right)=\mathrm{Protein}\;\mathrm{content}\;\mathrm{of}\;\\ \mathrm{diet}*\mathrm{Daily}\;\mathrm{feed}\;\mathrm{intake} \end{array}$$

Feces collected at T0 and T4 were dried in a forced-air oven and analyzed for nitrogen content (AOAC method 930.15; AOAC method 2001.11) (23). Protein content was calculated using 6.25 as a conversion factor (38). Apparent protein digestibility was assessed through an indirect marker analyzing the content of acid-insoluble ash (AIA) in both feed and feces (39). The relative digestibility of protein was calculated using the following formula:


$$\mathrm{ATTD}\;\left(\%\right)=100-100*\left(\frac{\begin{array}{l} \mathrm{Dietary}\;\mathrm{insoluble}\;\mathrm{acid}\;\mathrm{ash}\;\mathrm{content}*\\ \mathrm{protein}\;\mathrm{content}\;\mathrm{in}\;\mathrm{feces}\; \end{array}}{\begin{array}{l} \mathrm{Feces}\;\mathrm{insoluble}\;\mathrm{acid}\;\mathrm{ash}\;\mathrm{content}*\\ \mathrm{protein}\;\mathrm{content}\;\mathrm{in}\;\mathrm{diet} \end{array}}\right)$$

### 2.9 The antioxidant barrier of blood samples

To determine the serum antioxidant barrier, blood samples collected at T0, T2, and T4 were centrifuged at 3000 rpm for 15 min at 4°C. The obtained serum was analyzed through an Oxy-adsorbent test (Diacron, Grosseto, Italy) according to the manufacturer’s instructions. Absorbances were measured after 10 min of incubation at 37°C using a UV–Vis spectrophotometer (V630 UV–Vis, Jasco GmBH, Pfungstadt, Germany) at 546 nm.

The results were then expressed as μmol di HClO/mL according to the following formula:


HClOmL=Absblank−AbsSampleAbsBlank−AbsCalibrator∗calibrator

### 2.10 Statistical analysis

The data concerning the chemical and functional characterization of *A. nodosum*, *P. calcareum* and their combination were analysed using GraphPad Prism (version 9.0.0, Boston, MA, USA). The normality of the distribution of the data and residuals was evaluated by Shapiro–Wilk and D’Agostino–Pearson tests. For the total polyphenol and flavonoid content, and the antioxidant activity of the extract the data were analysed using one-way analysis of variance (ANOVA). For the growth inhibition assay, the data were analyzed using a two-way analysis of variance, which included the effects of treatment, time, and their interaction. As well, all the analyses concerning the functional characterization after the *in vitro* digestion were performed by applying the two-way analysis of variance. *Post hoc* pairwise comparisons were performed using Sidak’s test. Results derived from biological replicates were considered for statistical analysis of the *in vitro* assays. For the *in vivo* trial the number of animals (twelve for each group) was defined *a priori* using the GPower software, version 3.1.9.7., considering: (i) a power test of 80%, (ii) a protection level of 95%, (iii) an effect size of 0.5 (40–42). The effect size was defined according to previous studies that adopted similar experimental design for limiting the number of experimental animals according to the 3R principle (35, 43). Analyses were conducted using a repeated-measures two-way ANOVA. The results were evaluated using a full factorial model (Treatment: Trt, Time: Time, Interaction: Trt X Time). Diarrhea frequency was obtained by converting the fecal score data into a dichotomous variable (presence or absence) to evaluate observed frequencies through Pearson’s Chi-square test. For the *in vivo* trial, the individual animal constituted the experimental unit, so the number of replications was 12 for each group. The data are reported as the mean ± standard deviation (SD) or as mean ± standard error (SEM), and differences were statistically significant at *p* ≤ 0.05.

## 3 Results

### 3.1 Chemical characterization

Table 2 shows that both species tested had a high ash content, equal to 25.33 and 92.75%, respectively, for *A. nodosum* and *P. calcareum*. Specifically, analysis by ICP/MS allowed for the identification of the minerals’ profile present in the analyzed seaweeds (Table 2). In particular, the ICP/MS analysis disclosed that both *A. nodosum* and *P. calcareum* were rich in Strontium (Sr) (618.14 ± 8.166 mg/Kg and 2110.60 ± 33.640 mg/kg respectively), Iron (Fe) (407.94 ± 0.467 mg/Kg and 1004.90 ± 1.755 mg/Kg respectively) and Aluminum (Al) (211.28 ± 7.027 mg/Kg and 213.72 ± 6.386 mg/Kg respectively). Moreover, *A. nodosum* was mainly rich in Boron (B) and Manganese (Mn) (162.21 ± 1.613 mg/Kg and 100.80 ± 1.242 mg/kg respectively), while Titanium 48 (Ti 48) was particularly abundant in *L. calcareum* (456.87 ± 11.233 mg/Kg).

**Table 2:** Analyzed nutrient composition and mineral composition of *Ascophyllum nodosum* and *Lithothamnium calcareum*.

Nutritional composition (% as fed basis)		
	***Ascophyllum nodosum***	***Lithothamnium calcareum***
DM	91.44	99.60
Ash	25.33	92.75
*CF*	8.92	2.91
CP	6.93	0.21
EE	1.79	0.27

Mineral composition (mg/Kg as fed basis)		
	***Ascophyllum nodosum***	***Lithothamnium calcareum***
Aluminum (Al)	211.28 ± 7.027	213.72 ± 6.386
Antimony (Sb)	0.08 ± 0.005	0.10 ± 0.003
Arsenic (As)	40.06 ± 0.060	1.20 ± 0.068
Barium (Ba)	16.76 ± 2.459	6.92 ± 0.416
Boron (B)	162.21 ± 1.613	45.50 ± 2.075
Cadmium (Cd)	0.54 ± 0.011	0.56 ± 0.039
Calcium (Ca)	14.74 ± 1.720^*^	397.04 ± 14.487^*^
Chromium (Cr)	1.27 ± 0.154	4.53 ± 1.604
Cobalt (Co)	0.72 ± 0.022	0.57 ± 0.321
Copper (Cu)	2.01 ± 0.006	4.35 ± 1.511
Iron (Fe)	407.94 ± 0.467	1004.90 ± 1.755
Lead (Pb)	0.8 ± 0.237	0.1 ± 0.046
Manganese (Mn)	100.80 ± 1.242	83.66 ± 2.279
Magnesium (Mg)	7.77 ± 0.369^*^	30.00 ± 1.778^*^
Molybdenum (Mo)	0.72 ± 0.011	1.65 ± 0.658
Nickel (Ni)	3.10 ± 0.070	2.22 ± 0.349
Phosphorus (P)	0.65 ± 0.066^*^	0.47 ± 0.036^*^
Potassium (K)	21.62 ± 0.985^*^	0.20 ± 0.026^*^
Selenium (Se)	0.21 ± 0.103	0.66 ± 0.342
Silver (Ag)	0.07 ± 0.022	0.02 ± 0.007
Sodium (Na)	34.60 ± 1.616^*^	5.33 ± 0.352^*^
Strontium (Sr)	618.14 ± 8.166	2110.60 ± 33.640
Thallium (Tl)	Nd	Nd
Thorium (Th)	Nd	Nd
Titanium 47 (Ti 47)	7.96 ± 2.003	69.80 ± 0.433
Titanium 48 (Ti 48)	22.61 ± 2.751	456.87 ± 11.233
Uranium (U)	0.5 ± 0.007	2.7 ± 0.021
Vanadium (V)	2.93 ± 0.190	12.51 ± 0.046
Zinc (Zn)	31.51 ± 0.832	Nd

Nd, not determinable; *asterisked values are given in g/kg.

### 3.2 Functional characterization

#### 3.2.1 Evaluation of bioactive compounds

The evaluation of the Total Polyphenol Content (TPC) disclosed that *A. nodosum* had the highest concentration of TPC, equal to 330.42 ± 21.372 mg TAE/g of the sample, while *P. calcareum* showed a value of TPC equal to 11.45 ± 0.521 mg TAE/g of the sample (*p* < 0.001). A similar trend was also shown in the Total Flavonoid Content (TFC) evaluation. Obtained values were 213.85 ± 20.557 and 2.71 ± 0.900 mg CE/g of sample, respectively, for *A. nodosum* and *P. calcareum* (*p* < 0.001). Both assays showed values for the combination (1:1) of the two algae, which were half of those found for *A. nodosum*. Specifically, the TPC was found to be 140.42 ± 10.230 mg TAE/g of sample, while the TFC was found to be 107.07 ± 27.079 mg CE/g of sample.

#### 3.2.2 Evaluation of ABTS radical scavenging activity

The evaluation of the antioxidant activity of algae compounds showed that *A. nodosum* possessed higher antioxidant activity than *P. calcareum* (*p* < 0.001). In fact, the percentage of the inhibition of radical scavenging activity (PI%) of *A. nodosum* was 57.73 ± 1.444%, corresponding to 1313.22 ± 24.781 μMol Trolox eq/ g. On the other hand, the PI% of *P. calcareum* was of 2.96 ± 0.130%, which is equal to 20.07 ± 3.087 μMol Trolox eq/g. In addition, the extracts of the two algae placed in combination were analyzed to assess a possible synergistic effect. The results obtained showed that the PI% of the combination of the considered algae species is 48.85 ± 1.356%, resulting significantly higher (*p* = 0.0001) than that obtained by summing the inhibition rates of the algal extracts taken individually (45.10 ± 1.731%).

#### 3.2.3 Growth inhibition assay

**Figure 2:**
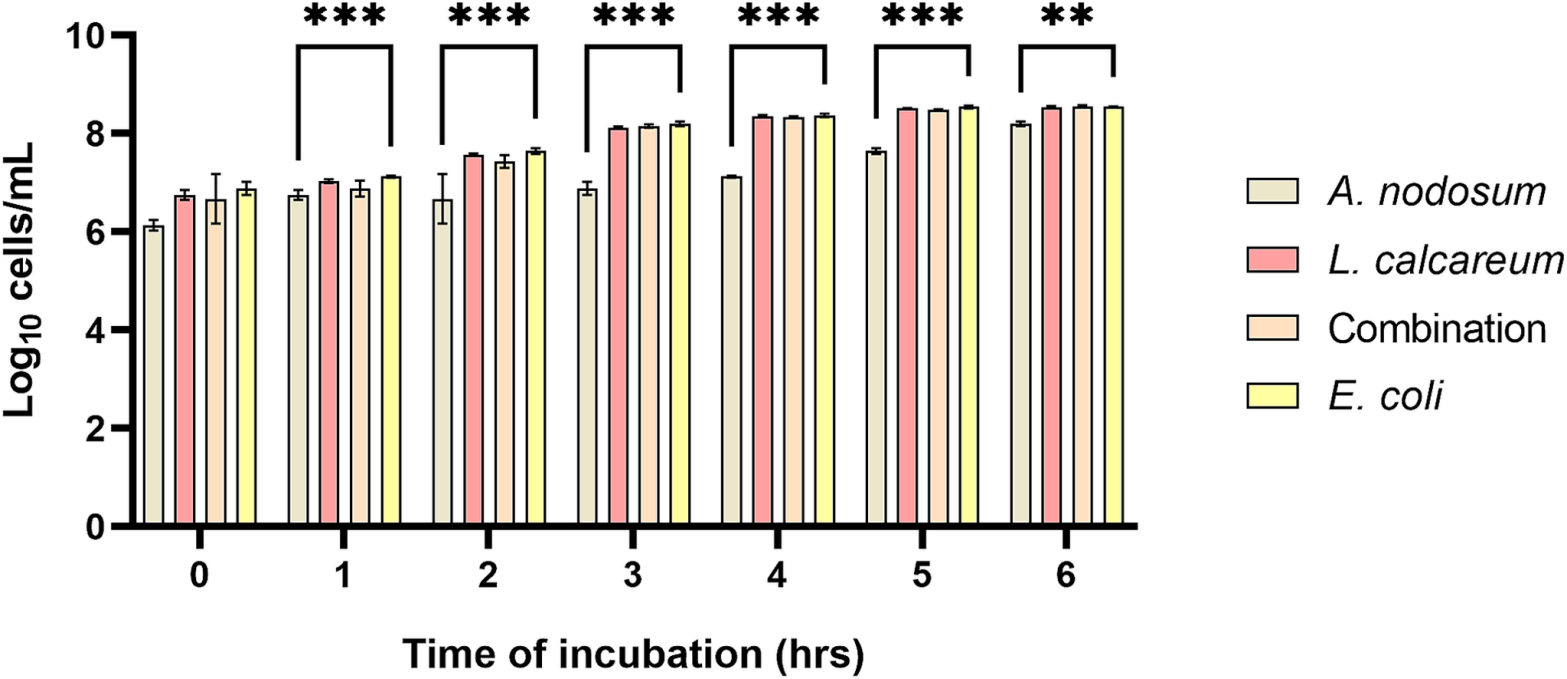
*Ascophyllum nodosum*, *Lithothamnium calcareum,* and their combination extract growth inhibition capacity against F4^+^ *E. coli*. The data are shown as the means and standard errors. Asterisks (*n* = 3) with different superscripts indicate significantly different means; **p = 0.002, ***p < 0.001. Time: *p* < 0.001; Treatment: *p* < 0.001; Time x Treatment: *p* < 0.001.

Growth inhibition was evaluated against F4^+^ *Escherichia coli*, belonging to bacterial strain collection of the Department of Veterinary Medicine and Animal Science at University of Milan (44). The results highlight that *A. nodosum* showed a higher growth inhibition capacity compared to both the combination and *P. calcareum*. In fact, it was observed that at a dilution of 1:8, starting from the first hour of incubation until the sixth hour of incubation, co-culture of *A. nodosum* with *E. coli* was able to lower significantly the growth of the microbial strain (*p* < 0.001) (Figure 2).

### 3.3 *In vitro* digestibility of algae and functional characterization after digestion process

#### 3.3.1 *In vitro* digestibility

After the *in vitro* simulation of digestion process *A. nodosum* disclosed a percentage of digestibility equal to 21.06 ± 0.142%, while *P. calcareum* highlights a higher digestibility, which has been around 44.16 ± 0.488%.

#### 3.3.2 Evaluation of bioactive compounds after simulated digestion

**Figure 3:**
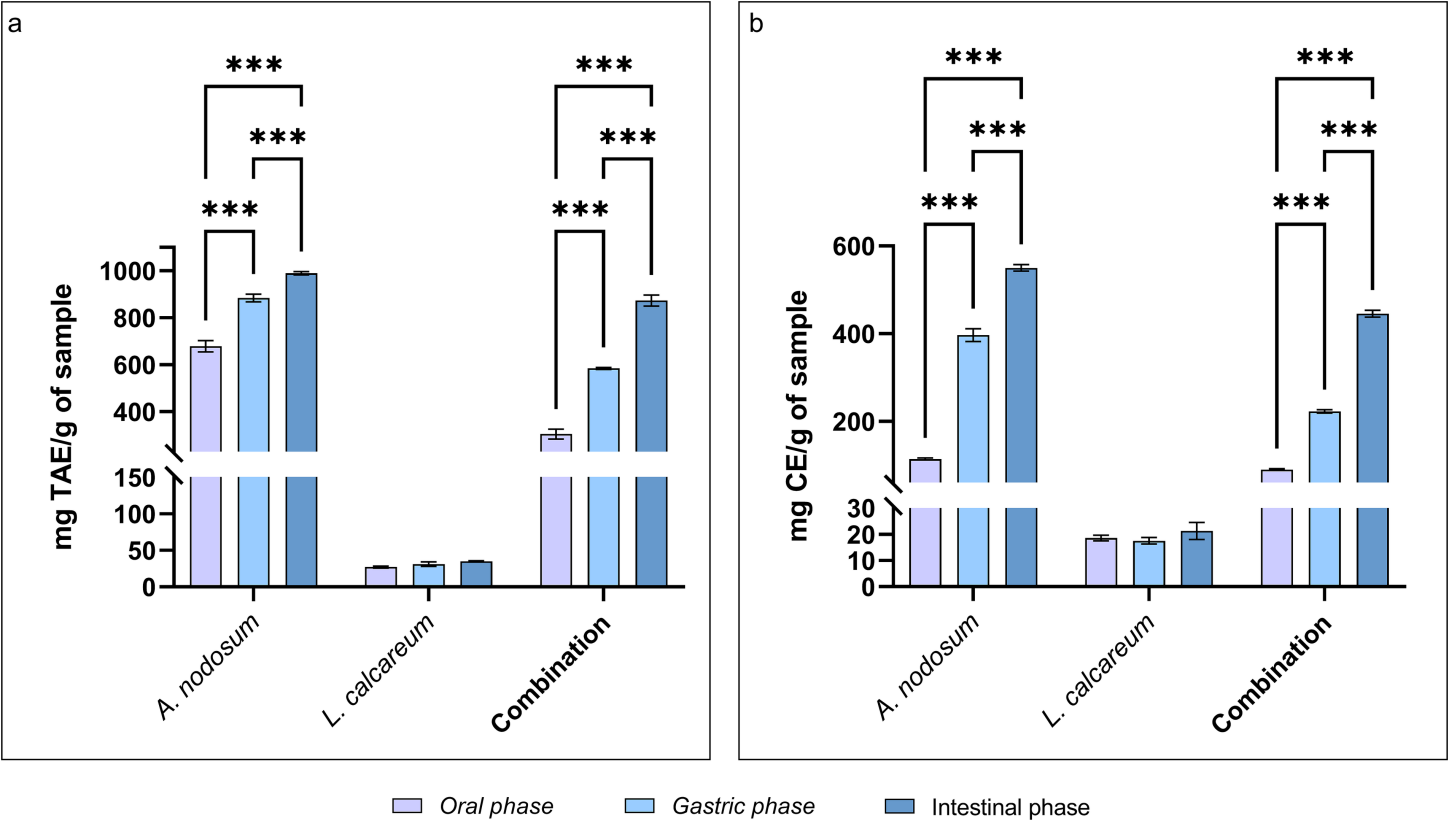
Phenolic and flavonoid compounds in *Ascophyllum nodosum*, *Lithothamnium calcareum* and their combination after the *in vitro* digestion. **(A)** Total phenolic content (TPC) after *in vitro* digestion. Time: *p* < 0.001; Treatment: *p* < 0.001; Time x Treatment: *p* < 0.001; **(B)** Total flavonoid content (TFC) after *in vitro* digestion. Time: *p* < 0.001; Treatment: *p* < 0.001; Time x Treatment: *p* < 0.001. TAE, tannic acid equivalent; CE, catechin equivalent. The data are shown as the means ± standard deviations (SDs) (*n* = 3). Asterisks (*n* = 3) with different superscripts are significantly different; ***p < 0.001.

The TPC and TFC were evaluated following the *in vitro* digestion process to assess the behavior of bioactive molecules following the digestive process. Our results, shown in Figure 3A, revealed that the polyphenol content increases as the digestive phases proceed. Specifically, after the oral phase, bioactive molecules of *A. nodosum* undergo a partial degradation that highlights a TPC content equal to 678.81 ± 53.684 mg TEA/g of sample. Following the subsequent digestive phases, the polyphenol content significantly increases to a value of 989.65 ± 16.968 mg TEA/g of sample after the intestinal phase (*p* < 0.001). As well as also for the combination of the algae species a significantly increasing trend was shown (*p* < 0.001), since starting from the end of the oral phase until the end of the intestinal phase the TPC is, respectively, 304.81 ± 46.089; 584.56 ± 9.391; 873.55 ± 52.294 mg TEA/g of sample. The evaluation of TPC of *P. calcareum* underlines that also in this case the TPC may slightly increase as the digestive process proceeds, although the difference is not statistically significant (*p* > 0.05). The same trend can be observed in flavonoids (Figure 3B). As highlighted from the polyphenol analysis during the first phase of digestion, the release of flavonoids was lower (113.78 ± 5.322 mg CE/g of sample for *A. nodosum*; 18.56 ± 2.634 mg CE/g of sample for *P. calcareum*; 89.63 ± 4.310 mg CE/g of sample for the combination), and then increase in the gastric and intestinal digestion phase (respectively: 396.61 ± 32.541 and 549.93 ± 16.856 mg CE/g of sample for *A. nodosum*, *p* < 0.05; 17.52 ± 3.040 and 21.26 ± 7.859 mg CE/g of sample for *P. calcareum*; 222.48 ± 8.490 and 445.31 ± 17.562 mg CE/g of sample for the combination, *p* < 0.05).

#### 3.3.3 Determination of functional activity after *in vitro* digestion

**Figure 4:**
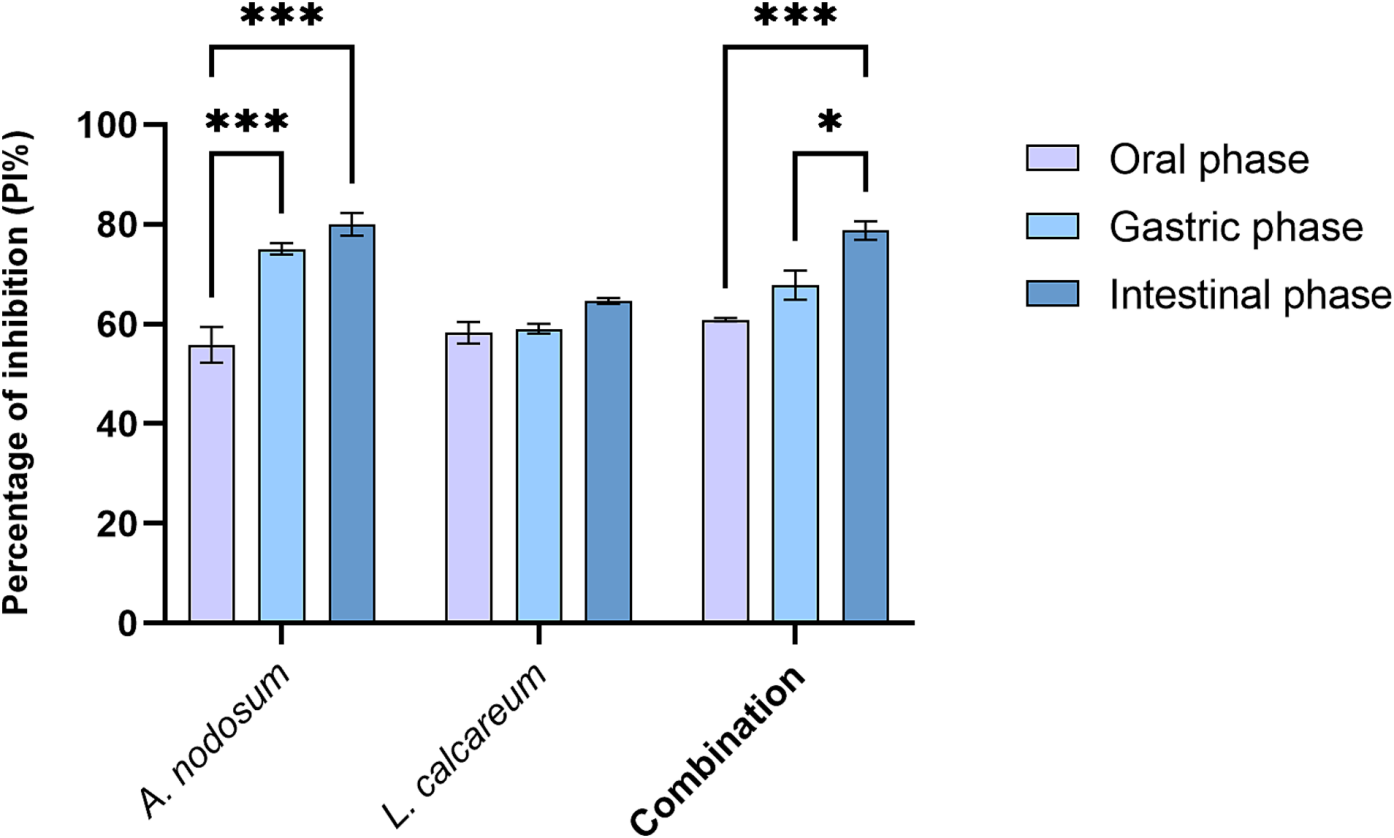
Percentage inhibition of radical scavenging activity (PI%) after *in vitro* digestion of *Ascophyllum nodosum*, *Lithothamnium calcareum,* and their combination. The data are shown as the means ± standard deviations (SDs) (*n* = 3). Asterisks (*n* = 3) with different superscripts are significantly different; ***p < 0.001; *p = 0.04. Time: *p* < 0.001; Treatment: *p* < 0.001; Time x Treatment: *p* = 0.001.

**Figure 5:**
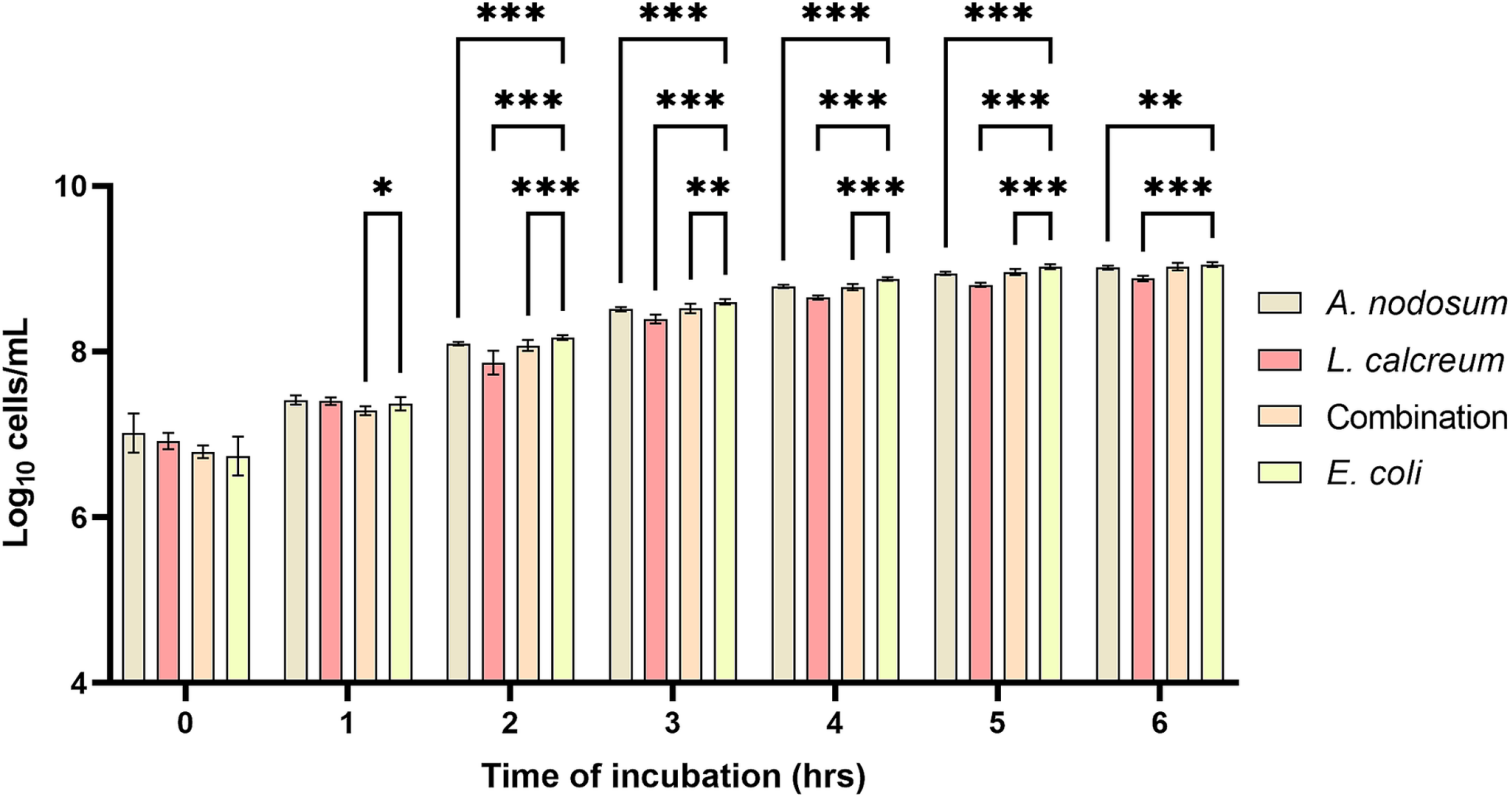
Growth inhibition of *Ascophyllum nodosum*, *Lithothamnium calcareum* and their combination against *E. coli* F4+ after the digestive process. The data are shown as the means ± standard deviations (SDs) (*n* = 3). Asterisks (*n* = 3) with different superscripts are significantly different; ***p < 0.001, **p < 0.004, *p = 0.02. Time: *p* < 0.001; Treatment: *p* < 0.001; Time x Treatment: *p* < 0.001; Subject: *p* = 0.007.

Regarding the functional activity of the selected algal species the antioxidant activity and the growth inhibitory activity against F4^+^ *E. coli* were evaluated. Concerning the antioxidant activity, as shown in Figure 4, the percentage of inhibition increases proceeding with the digestive process for all the samples tested. In particular this increase was statistically significant for *A. nodosum* (*p* < 0.001) and for the combination (*p* < 0.04). As well as the algal extracts show an ability to inhibit the growth of F4^+^ *E. coli* this property remains even after the digestive process. In fact, both algal species considered and their combination when co-cultured with F4+ *E. coli* after undergoing the *in vitro* digestion process are able to significantly slow down its proliferation. As shown in Figure 5, it can be observed that from the second hour of incubation, the reduction in microbial growth for *A. nodosum, P. calcareum* and their combination.

### 3.4 Evaluation of experimental diets composition

Experimental diet evaluation of principal nutrient content revealed that nutrient concentrations are in line with NRC guidelines (45), thus fulfilling the nutritional requirements of weaned piglets. The inclusion of algae species did not influence the nutrient profile of treatment groups (Table 3).

**Table 3:** Analyzed nutrient composition of experimental diet.

Nutrient (%)	Experimental diet	
	CTRL	ALGAE
DM	93.19 ± 0.050	93.38 ± 0.042
CP	18.38 ± 1.421	18.35 ± 0.707
EE	4.51 ± 0.042	4.63 ± 0.035
CF	2.74 ± 0.161	2.66 ± 0.026
Ash	4.82 ± 0.239	4.89 ± 0.135
Starch	31.19 ± 0.832	30.81 ± 0.818

Data are shown as the means ± standard deviations (SDs) (*n* = 3). DM, dry matter; CP, crude protein; EE, ether extract; CF, crude fiber.

### 3.5 Zootechnical performance

**Figure 6:**
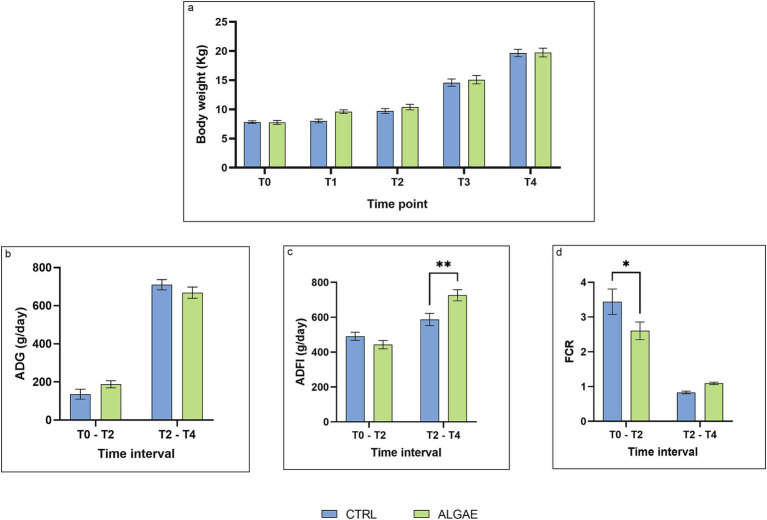
Zootechnical performance from T0 to T4. **(A)** Body weight at T0, T1, T2, T3, T4. Time: *p* = 0.0903; Treatment: *p* < 0.001; Time x Treatment: *p* = 0.5326; **(B)** Average Daily Gain (ADG) in the time interval T0 - T2 and T2 - T4. Time: *p* = 0.8631; Treatment: *p* < 0.001; Time x Treatment: *p* = 0.0426; Subject: *p* = 0.0914; **(C)** Average Daily Feed Intake (ADFI) in the time interval T0 - T2 and T2 - T4. Time: *p* = 0.2105; Treatment: *p* < 0.001; Time x Treatment: *p* < 0.002; Subject: *p* = 0.011; **(D)** Feed Conversion Rate (FCR) in the time interval T0 - T2 and T2 - T4. Time: *p* = 0.1860; Treatment: *p* < 0.001; Time x Treatment: *p* = 0.0152. The data are shown as the means ± standard error (SEM). Asterisks with different superscripts are significantly different; **p = 0.003, *p = 0.0166.

The individual Body Weigh (BW) recorded weekly showed no significant differences throughout the experimental period (Figure 6A). Similarly, the Average Daily Gain (ADG) also showed no significant differences between the two experimental groups. In particular in the first part of the trial the ADG of the CTRL group was 135.70 ± 26.249 g/day and those of the ALGAE group was 187.50 ± 18.736 g/day, while in the second part of the trial the ADG was of 710.10 ± 26.862 g/day and 668.50 ± 29.377 g/day, respectively for the CRTL and ALGAE group. During the second part of the trial, the Average Daily Feed Intake (ADFI) of the CTRL group was significantly lower than that of the ALGAE group (587.50 ± 34.883 g/day and 726.20 ± 32.152 g/day, respectively; *p* = 0.0030). Finally, the Feed Conversion Ratio in the first half of the trial the control group has a statistically higher conversion index than the ALGAE group (3.44 ± 0.364 and 2.60 ± 0.254, respectively; *p* = 0.016), while in the second half of the trial both conversion indices decreased without showing a significant difference between the two groups (Figures 6B–D).

### 3.6 Diarrhea occurrence

Considering the entire duration of the trial, the cases of diarrhea that occurred were the same for both the CTRL and ALGAE groups. Notably, cases of diarrhea were only observed in the first period of the trial where for both experimental groups the frequency of observed diarrhea was 3.85% out of the total number of observations. On the other hand, during the second period of the trial no cases of diarrhea were observed in either group.

### 3.7 Microbiological evaluation of fecal samples

**Figure 7:**
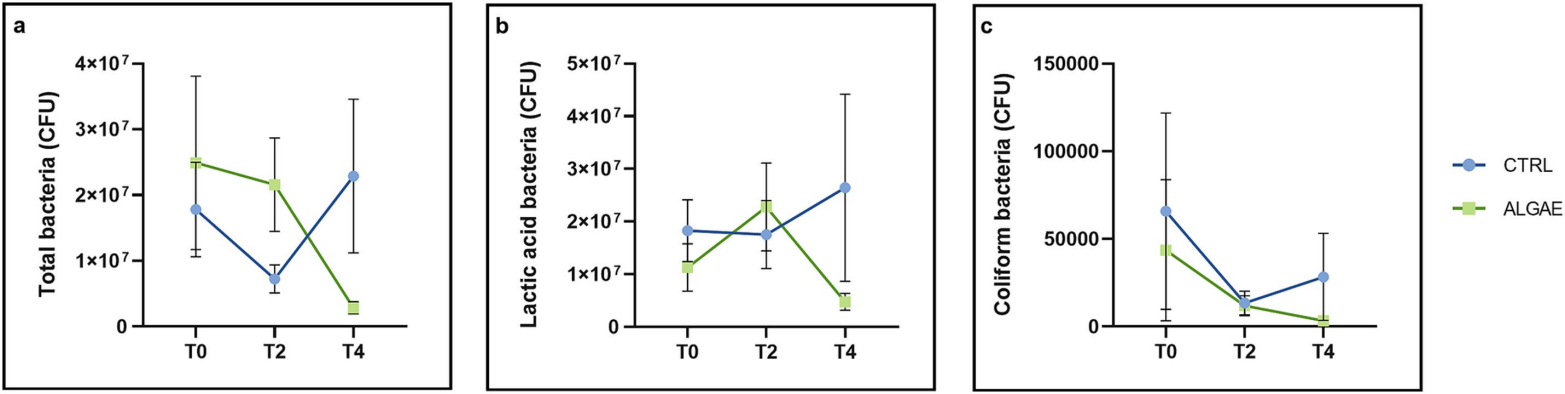
Microbiological analysis of feces. **(A)** Microbiological evaluation of total bacteria on PCA medium. Time: *p* = 0.9419; Treatment: *p* = 0.5631; Time x Treatment: *p* = 0.0697; **(B)** Microbiological evaluation of lactic acid bacteria on MRS medium. Time: *p* = 0.0838; Treatment: *p* = 0.8488; Time x Treatment: *p* = 0.8823; **(C)** Microbiological evaluation of coliform bacteria on VRBL medium. Time: *p* = 0.0432; Treatment: *p* = 0.0769; Time x Treatment: *p* = 0.5892. The data are shown as the means ± standard error (SEM).

Bacterial counts were performed on fecal samples for the determination of colony-forming units (CFU) of total, lactic, and coliform bacteria. The results obtained from the microbiological analysis of the feces (Figure 7A) showed that in both groups at the initial time (T0) the total bacteria present amounted to 2.14×10^7^ ± 5.031×10^6^ CFU/g, of these the majority were lactic acid bacteria (1.82×10^7^ ± 2.038×10^7^ CFU/g), while coliforms were present in a reason of 0.5% of the total. At T2 it is possible to observe that in both groups there was a 22% reduction in coliform bacteria count (Figure 7C). With regard to the evaluation of lactic acid bacteria at the same time point, it is possible to find, from the data obtained (Figure 7B), the administration of the feed fortified with algae allowed an increase in this bacterial genus, which was found to be present in an amount of CFU/g equal to 2.28×10^7^ ± 4.070×10^7^ in the ALGAE group versus 1.75×10^7^ ± 3.102×10^7^ CFU/g in the CTRL group. At the final time (T4), it is possible to observe that the coliform bacteria count of the ALGAE group is lower, although not significantly (*p* > 0.05), compared with the count at T2 (3.28×10^3^ ± 9.936×10^3^ CFU/g vs. 1.19×10^4^ ± 2.274×10^4^ CFU/g, respectively).

### 3.8 Protein intake and apparent protein digestibility

The calculation of protein intake revealed that during the first period of the trial, the protein intake did not differ significantly between the two experimental groups (CTRL: 85.93 ± 14.123 g/day; ALGAE: 77.50 ± 14.470 g/day; *p* > 0.05). On the other hand during the second period of the trial the protein intake of the ALGAE group (127.08 ± 19.491 g/day) was significantly higher than the CTRL group (102.81 ± 21.147 g/day) (*p* = 0.008). The nitrogen content of fecal sample revealed no significant differences between the experimental group compared to the CTRL group (5.20 ± 1.201% for CTRL and 3.35 ± 0.212% for ALGAE group, on dry matter basis) at T4. These results were also reflected in the evaluation of the apparent protein digestibility that, as well as, revealed no statistical difference between the CTRL and the ALGAE groups at T4 (87.89 ± 3.110% and 88.01 ± 1.745% respectively).

### 3.9 The antioxidant barrier of blood samples

**Figure 8:**
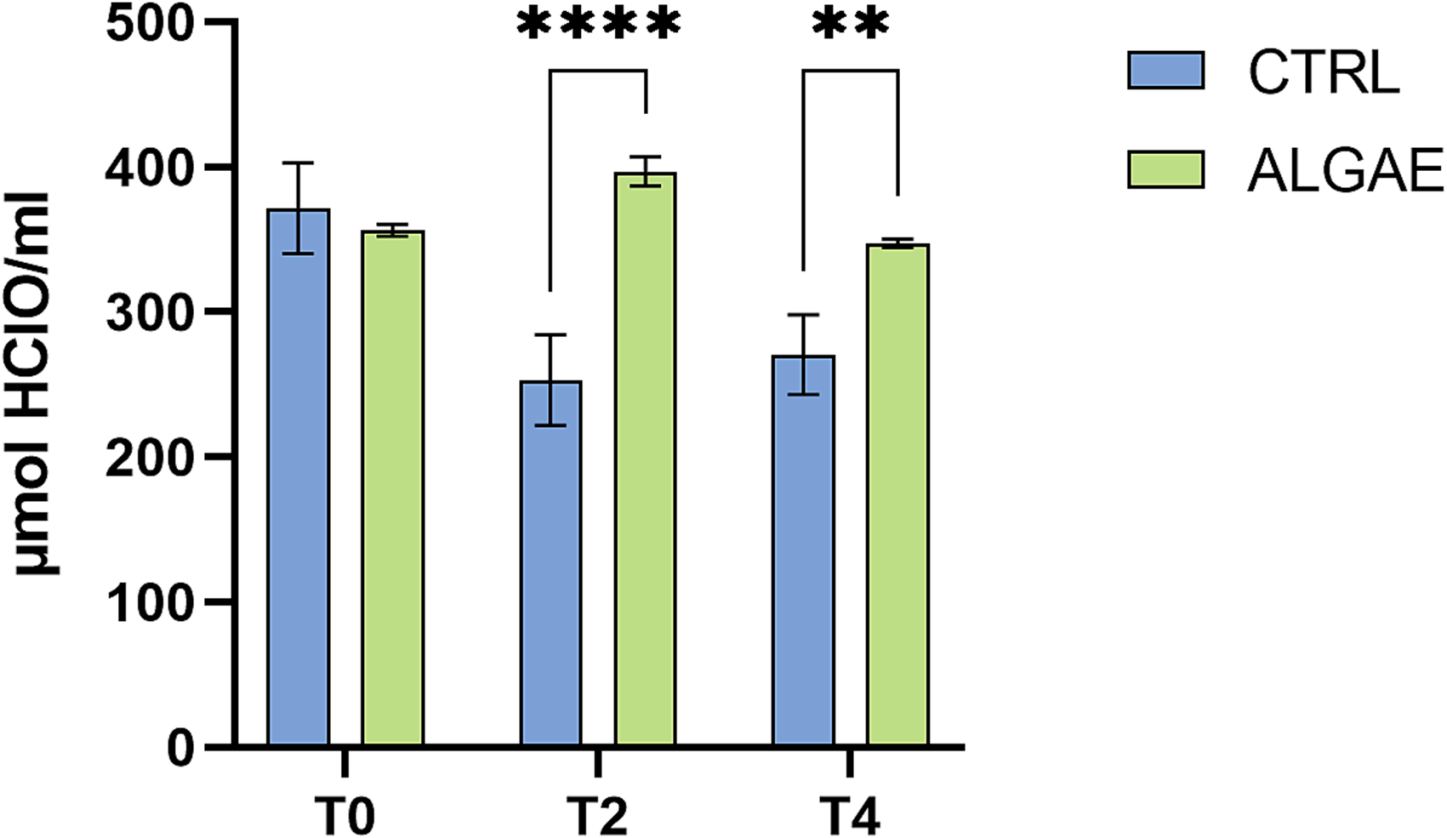
Serum values of Oxy Adsorbent test measured at T0, T2, and T4 of trial in CTRL and ALGAE groups. The data are shown as the means ± standard error (SEM). Asterisks with different superscripts are significantly different; ****p < 0.0001, **p < 0.001. Time: *p* < 0.001; Treatment: *p* = 0.003; Time x Treatment: *p* < 0.001.

The oxidative status of the serum barrier was evaluated as the ability of the latter to oppose the oxidizing action of a hypochlorous acid (HClO) solution using the OXY adsorbent test. The results obtained showed that the addition of *A. nodosum* and *P. calcareum* within the diet fed to post-weaning pigs is able to bring about an improvement in serum barrier antioxidant activity. In fact, comparing the CTRL group with the ALGAE group, it can be observed that at both T2 and T4 the serum barrier antioxidant activity values of the ALGAE group are 396.83 ± 10.125 and 347.35 ± 2.892 μmol of HClO/mL, respectively, resulting significantly higher (*p* < 0.010) than those obtained at the same time points in the CTRL group (253.10 ± 31.364 and 270.61 ± 27.439 μmol of HClO/mL, respectively) (Figure 8).

## 4 Discussion

In recent years, there has been a growing interest in finding functional and sustainable ingredients that can be used in animal nutrition to promote health, reduce the use of antibiotics, and ensure the profitability of farms (46). This aspect is emphasized in pig farming, where numerous multifactorial pathologies occur during weaning, and nutrition plays a central role (47). On this aspect, the combination of multiple functional ingredients that can increase their individual spectrum of action has emerged as an interesting approach. The broad spectrum can indeed be related not only to synergistic effects but also to the different mechanisms of action targeting various pathways. By combining functional ingredients with diverse modes of action, such as immune modulation, gut health promotion, antioxidant activity, and anti-inflammatory effects, the overall impact on animal health and performance can be amplified. This comprehensive approach addresses multiple aspects of animal physiology and metabolism, resulting in a more holistic and effective nutritional strategy (48, 49).

With these assumptions, our study is built upon the promising findings reported by Frazzini et al. (22), where the combination of *A. nodosum* and *P. calcareum* demonstrated synergistic activity in inhibiting pathogenic bacterial strain of *E. coli* and antioxidant properties *in vitro*. Particularly, after testing the conservation of functional properties following the simulated *in vitro* digestive process, we aimed to test the combination of algae *in vivo* to gain a comprehensive understanding of the biological effects of functional ingredients in animals and to inform evidence-based practices in animal nutrition and health management.

### 4.1 Chemical characterization

The chemical composition of algae should be taken into account to maintain the nutrient balance of the experimental diet. Despite their low percentage of inclusion, algae may not significantly influence the total percentage of macronutrients in the diet. However, their influence on micronutrients can be crucial.

The chemical evaluation of *A. nodosum* and *P. calcareum* showed results in line with the scientific literature once again highlighting the high mineral content present in these algal species (50). The high calcium (Ca) content of *P. calcareum* (51) requires careful evaluation at the time of inclusion in the diet. In fact, when formulating diets, in addition to the most important nutritional components, specific ratios must also be taken into account, including the calcium-phosphorus ratio. If this ratio is not maintained correctly, a series of disturbances are created at the metabolic pathway level that have important repercussions on animal health (52, 53). In fact, as reported by Sun M. et al. (54), imbalances in the metabolism of calcium and phosphorus can lead to skeletal and cardiovascular diseases. *A. nodosum* also displayed a high mineral content. Still, the attention paid to this alga at a nutritional level is due to its protein composition, which is mostly of high nutritional value (55). Although the total mineral content of both algae is high the analyses conducted in ICP-MS ensured that those elements considered undesirable in animal nutrition such as arsenic (As), cadmium (Cd), lead (Pb), cobalt (Co), and molybdenum (Mo) did not exceed the threshold levels for feed established by regulation suggestion the lack of toxic risk for feed formulation considering low percentages of seaweeds inclusion. Likewise, Iron (Fe), manganese (Mn), and selenium (Se) did not exceed the threshold limits in swine nutrition (56, 57). Additionally, great importance has been placed on dietary zinc levels. Zinc (Zn) is indeed an essential micronutrient required for proper growth and development of the animal as it is involved in several structural and biological functions, such as enzymatic reactions, DNA and RNA metabolism, protein synthesis, gene expression, cell proliferation and differentiation and cell-mediated immunity (58, 59). From June 2022, the maximal allowed dietary Zn level for weaned piglets in the EU is 150 mg/kg diet (60, 61). Analyses have shown that the algae used have low levels of zinc, which would therefore not unduly influence the legal limit for a complete feed, particularly when they are included in the feed as functional ingredients and therefore with an inclusion rate of no more than 2%.

### 4.2 Functional characterization

The undeniable nutritional characteristics of algae are also associated with good functional properties due to the presence of various bioactive compounds (22, 62–64). *A. nodosum* and *P. calcareum* are both marine algae rich in bioactive compounds, including flavonoids and polyphenols (65). This molecule class is a group of plant pigments that have been shown to have a variety of health benefits, including antioxidant and anti-inflammatory properties, protecting against cell damage, and improving blood vessels’ health (66, 67).

*A. nodosum* stands out in the algae kingdom due to its impressive content of total polyphenols and flavonoids (68). While total phenols comprise a broader range of antioxidant compounds, flavonoids represent a specific subclass known for their health benefits. The presence of different bioactive compounds and the molecular interaction among them is particularly interesting since the high total phenolic content, attributable to the content of phenols, including phlorotannins, and the presence of laminarin and fucoidan (69, 70) suggests a strong antioxidant capacity, potentially reducing free radical damage and inflammation (71, 72). Moreover, the functional components of brown algae, such as phlorotannins, which are also known to have bacteriostatic and bactericidal activity, can contribute to the antimicrobial properties highlighted in the study (73, 74).

On the other hand, being *P. calcareum* mainly recognized for its mineral component, particularly calcium, magnesium, and carbonate, research has currently focused on these minerals and their potential benefits and they are lacking in investigating the bioactive properties of this algae (75, 76). Analyses conducted in this study on the matrix of *P. calcareum* showed a, albeit minimal, polyphenols and flavonoids content. The presence of different bioactive molecule could explain the synergistic effect revealed by the analysis of the functional properties of the combination of the two algal extracts as demonstrated in a previous work (22).

In fact, has been demonstrated that the combination of different antioxidant sources could enhance their effect on radical scavenging activity (71, 72, 77). Moreover, *P. calcareum* extract was found to be able to reduce the growth of F4+ *E. coli*, thus probably due to the ability of red algae to produce antimicrobial metabolites, such as diterpenoids (78), monoterpenes (79), phenolic compounds (80), sterols (81), polysaccharides (82), and fatty acids (83).

### 4.3 Functional characterization after *in vitro* digestion

*In vitro* digestion models mimic the conditions of the gastrointestinal tract, including the stomach and intestine, by allowing the theoretical determination of the digestibility of a given ingredient. Thus, helping to evaluate the inclusion of specific ingredients within the diet. Algae, as reported in the literature, are not always extremely digestible; this depends on the species to which they belong. Our results were in line with this statement. In fact, our findings revealed that *A. nodosum* had a low digestibility, probably due to the high phlorotannin content (84, 85), which, as reported by Ford et al. (86), significantly affects the digestibility of this seaweed. Therefore, the inclusion of poorly digestible foods, as in the case of *A. nodosum* must be carefully considered when formulating the diet where it is advisable to include such ingredients in not too high percentages that consequently do not affect digestibility. In the case of high inclusions of ingredients rich in phlorotannin, such as *A. nodosum* it is also possible to introduce within the feed additives such as polyethylene glycol and polyvinylpyrrolidone that make the phlorotannin themselves more digestible (87). On the other hand, *P. calcareum* revealed a higher digestibility, probably due to its mineral composition, which showed a higher solubility of this alga than other limestone sources, suggesting better digestibility and also higher bioavailability of calcium when used in animal diets (88). However, its digestibility, such as for *A. nodosum* is influenced by other factors such as the presence of phenolic compounds, which can inversely affect protein digestibility, and the structural characteristics of seaweeds, play a significant role in their overall nutritional quality (89). The *in vitro* digestion models allowing also to simulate how functional ingredients maintain their activities after digestion. This is essential for understanding how functional ingredients behave within the gastrointestinal tract and predicting their physiological effects, thereby informing the development of effective and bioavailable products for promoting health and welfare.

The interest in bioactive molecules from algae has surged in recent years due to their potential health benefits and applications in various industries. Algae, both macroalgae and microalgae, are rich in diverse bioactive compounds that exhibit a wide range of biological activities, including antioxidant, anticancer, anti-inflammatory, and immune-modulating effects (90, 91). Therefore, it is also important to focus attention on the knowledge of bioactive compounds bioavailability, and their modification after the digestion process. The results obtained disclose that the total phenolic content and total flavonoid content of *A. nodosum* and their combination showed a gradual and significant increase during *in vitro* digestion. This is due to the fact that, initially, during the oral phase, only simple phenolics, such as phlorotannin, gallic acid, protocatechuic acid, and 4-hydroxybenzoic acid, are released due to the short digestion time (92). Once in the gastric phase, the decrease of pH and the presence of pepsin enzyme allowed the degradation, oxidation, and polymerization of a higher amount of phenolic compounds (93). Finally, when, in the intestinal phase, the samples come into contact with an alkaline environment and with the presence of pancreatic enzymes, a complete release of the polyphenolic component occurs. This is due to the fact that a neutral pH allows the release of those polyphenolic molecules that are instable in an acidic environment, and it is also reported that the presence of pancreatin affects the binding of the polyphenolic molecules with the other molecules in the food matrix, resulting in the release of this molecular class (94). Moreover, in addition to the different environments of the three digestive phases, the polyphenol content released, as reported by Wang and colleagues (95), is also related to chemical compositions, dietary fiber, hydrophobic interactions, hydrogen bonding, and covalent bonds of the digestive matrix. At the same time as an increase in bioactive components was observed during the different digestion steps, the relative antioxidant activity also showed a significant increase (*p* < 0.05) as digestion progressed. This behavior could be due, as well as for the bioactive molecules, to pH variation during *in vitro* digestion. In fact, the transition from the acid medium to the alkaline favors the release of phenols and flavonoids which are correlated to the antioxidant activity (96, 97). Additionally, the antioxidant capacity is further linked to the interplay of phenolic compounds with other substances, such as minerals or dietary fiber, released during the digestion process. These substances in which algae are rich can influence solubility and the availability of phenols. In any case, in the interpretation of these results, we have to take into account that during the digestion process other non-antioxidant food components, such as amino acids, sugars, and uronic acids, can be released and these might interfere with the Trolox equivalent antioxidant capacity assays increasing the values of antioxidant activity observed (98). Finally, as reported by Chen et al. (99), polyphenols, thanks to their molecular structure, are able to develop antimicrobial activity. Gram-negative bacteria, such as *E. coli*, are more resistant to the antibacterial activity of phenolic compounds thanks to the high levels of phospholipids on the lipophilic outer membrane (100). Therefore, the antibacterial mechanism operates through the buildup of hydroxylic groups within lipid bilayers, disrupting the interaction of lipoproteins and enhancing the permeability of the cell membrane (101). In any case, these findings need cautious interpretation because is important to note that polyphenols and other antioxidant and antimicrobial compounds may undergo metabolism by gut microbiota (102). Therefore, the results obtainable through the *in vitro* digestion process may not fully reflect what happens in a living organism’s intestinal system.

### 4.4 Zootechnical performance

In pig farming, the weight that the animal reaches during the weaning phase is fundamental, as it has a knock-on effect on the entire production cycle of pigs. In fact, higher weights at weaning will lead to an earlier attainment of slaughter weight, reflecting positively on the income of the farmer (103). A higher weight also translates into better intestinal health, consequently improving the performance of the animal (104). The study carried out showed that the inclusion of *A. nodosum* and *P. calcareum* in the diet of post-weaning pigs did not have a depressive effect on the growth of the animals, maintaining the growth curve unchanged, and consequently also the average daily weight gain, between the CTRL group and the ALGAE group. Although there are currently no studies in the literature evaluating the inclusion of the chosen algal combination in the diet of post-weaning piglets, the data obtained are in line with other studies considering the inclusion of individual algal species. Michiels et al. (105) following the administration of three different amounts (2.5, 5.0 or 10.0 g of dried seaweed per kg) of *A. nodosum* in 21-day-old piglets observed no significant differences between the growth curves of the control and treatment groups. On the other hand, the administration of 3 g/kg; 6 g/kg; 9 g/kg of *A. nodosum* in post weaning piglets for 28 days disclosed that daily gain, and carcass weight were reduced in a linear manner with the inclusion of increasing levels of the brown seaweed (106). Even at higher doses of inclusions of *A. nodosum* (20 g seaweed kg-1 of feed) no significant increases in weight of the animals in the treatment group were found (107). Furthermore, the data obtained suggest that in the first part of the trial, feed consumption did not vary between the two groups, emphasizing that the introduction of the algae did not impact the palatability of the feed. This is of great importance, because food intake is crucial both for ensuring body growth but also for ensuring the integrity of the intestinal mucosa. In fact, is reported that maintenance of nutrition after weaning would prevent the normal decline in villous height and increase in crypt depth and hence preserve the structure and function of the small intestine, consequently preserving intestinal health (108, 109). Nevertheless, as the animals increased in age, and particularly from the 49th day of age, an increase in food consumption was observed, the introduction of the algae may possibly have had a positive effect and potentially a difference in weight could be observed over longer experimental periods (110). Finally, a significant difference in the FCR value could be observed during the test period. This may suggest that the inclusion of a small amount of algae may have had a negative impact on feed efficiency as the animals had to adapt to a new diet with a different fibrous composition. In fact, many studies report that the ‘recalcitrant’ cell wall of algae makes the digestion of some of their compounds more difficult, often suggesting treatments before feeding them to monogastric (111–113). Nevertheless, the animals reached a similar weight to the CTRL group while maintaining a growth rate that did not differ significantly.

### 4.5 Microbiological evaluation of fecal samples

If, on the one hand, the inclusion of algae in the diet did not influence the zootechnical performance, as well as the fecal score and consequently the diarrhea frequency that was unvaried between the two experimental groups. On the other hand, it revealed potential beneficial effects on intestinal bacterial populations. At T2, a reduction in coliform bacteria was observed in both groups. This shows that, in the time between T0 and T2, the experimental animals, regardless of the diet they received, achieved physiological well-being, which allowed them to be less susceptible to coliform infections, which are known to be the most common in stressful phases of pigs (8). As for lactic acid bacteria, at the same time point, it is possible to find that the administration of feed supplemented with algae has allowed an increase in this type of bacteria, which is known to be beneficial for the intestinal health of pigs (114). In fact, several studies report that these microorganisms help with the digestion of nutrients, provide mucosal protection and fight other pathogenic bacteria, including coliforms (115). Moreover, the greater the amount of beneficial bacteria in piglets microbiota, the lower the chance that they will develop diarrhea, given that the concentration of lactobacilli is directly proportional to good intestinal health (116). Huang et al. (117) reported that lactobacilli improved microbial balance in the digestive tract of piglets during the first 2 weeks after weaning and resulted in fewer cases of diarrhea and an increase in growth. Supporting the beneficial effect of algae feeding in the post-weaning piglet diet different studies report that the compounds present in the algae, particularly in brown seaweeds stimulated the growth of Lactobacilli (118, 119), and reduced the enterobacteria population or *Escherichia coli* (120, 121). Dierick et al. (107) revealed that the administration of *A. nodosum* (10 g kg − 1) in weaned piglets had a reducing effect on the *E. coli* load in the stomach and small intestine, while the lactobacilli/*E. coli* ratio was enhanced in the small intestine, indicating a beneficial shift in the microbial population. Other studies report also that the administration of extracts of bioactive components of algae, such as laminarin and fucoidan, lead to an increase in *Lactobacillus* spp. and a reduction of *E. coli* in swine feces (122–124). In conclusion, our data showed the positive potential of algae, but an evaluation of the entire microbiota is probably necessary in order to assess the overall effect of this innovative combination of algae in weaned piglets.

### 4.6 Protein intake and apparent protein digestibility

Our results regarding protein intake are in line with what was shown in the evaluation of feed intake, which was found to be higher in the ALGAE group during the second period of the trial. Despite this, the analysis of the fecal composition disclosed that the supplementation of algae within the diet did not affect the digestibility of the protein component as well as the nitrogen utilization. This is probably due to the inclusion of seaweed, which rich in polyphenols reduces protein digestibility (125, 126). However, the present study proved that using low levels of seaweed inclusion in the diet did not negatively affect the protein digestibility of the diet in post-weaning pigs. Although feces-based apparent digestibility analysis is an important parameter for estimating nutrient utilization efficiency using a minimally invasive method, few studies to date report the impact of algae on diet digestibility. However, our results are in line with those of Czech and colleagues who did not register significant differences in crude protein digestibility, supplementing 0.6 and 1% of *A. nodosum* from 18 to 64 days of age in weaned piglets (127). As well as another study focused on the supplementation of 0.05, 0.1, and 0.15% of *Ecklonia cava* in postweaning pigs showed no significant differences in the crude protein digestibility at 14 and 28 days after weaning (120). Although it is known that the inclusion of seaweed in the diet can slow down the digestion and absorption of nutrients (127), our results can be explained by the presence in seaweed of several antioxidant compounds that could protect the digestive tract from oxidative damage, improving intestinal health and digestive processes (128).

### 4.7 Evaluation of serum antioxidant barrier of blood samples

Many factors, such as physiological, environmental, and dietary, can induce the host to produce a large number of free radicals, which can cause oxidative damage in pigs that is often accompanied by other pathological factors, which have a direct negative impact on pig performance and healthy growth.

Nowadays, the higher pressure due to maximizing production in pig farming is one of the causes of the phenomenon of oxidative stress, a condition harmful to pig health and leading to higher feed costs. Therefore, developing effective methods to reduce oxidative stress is becoming essential. Since, under oxidative stress, body nutrient metabolism direction changes, current research efforts are primarily focused on alleviating oxidative stress in pigs by supplementing diets with additional supplementation of different nutrients or non-nutritional additives (129–131). Our study highlighted that the inclusion of *A. nodosum* and *P. calcareum* in the diet administered to post-weaning pigs is also able to improve the antioxidant activity of the serum barrier thereby protecting cells from damage caused by free radicals, probably thanks to the bioactive compounds with antioxidant capacity present in the algae considered. This result is supported by several studies in the literature where polyphenols were found to be attractive feed additives for nutritional management in pig production with the aim of mitigating oxidative stress in animals (132–134). Additionally, the study carried out by Czech and colleagues (127) highlights that the supplementation of *A. nodosum* can bring improvements in the oxidative state, since evidence were found in the reduction in lipid peroxidation markers and increased activity of redox enzymes in various tissues.

## 5 Conclusion

The present work first investigated the chemical and functional properties of two algal species *A. nodosum* and *P. calcareum*. Our study disclosed that the algae considered possess strong antioxidant and antimicrobial capabilities and that these improve once the algae are put in combination suggesting a possible synergistic effect given by the interaction of the biomolecules present. Moreover, it is also highlighted that the functional properties of *A. nodosum* and *P. calcareum* were maintained during the digestion process, further reinforcing the beneficial effects of introducing this ingredient within the diet. Therefore, was decided to evaluate the inclusion of the combination of the two algal species in the diet of post-weaning piglets. This part of the study showed that *A. nodosum* and *P. calcareum* within the feed did not alter zootechnical performance and increades the feed intake during the last week of trial. However, their inclusion highlighted the bioactive properties of these algal species. The group that consumed algae in their feed showed a stronger serum antioxidant barrier. Moreover, including algae led to a slight reduction in the presence of coliform bacteria. The results obtained in this study, although preliminary, also confirmed the bioactive potential of the algal species considered *in vivo*. Therefore, this study may represent a good starting point to investigate the bioactive properties of algae further in order to include them in animal nutrition to reduce the use of antibiotics by preventing the colonization of pathogenic bacterial strains during the most critical stages of pig farming.

## Data Availability

The original contributions presented in the study are included in the article/Supplementary material, further inquiries can be directed to the corresponding author.
