# Supplementary material for: Chemical-functional characterization of Ascophyllum nodosum and Phymatolithon calcareum and dietary supplementation in post-weaning pigs
Source: Front Vet Sci. 2024 Dec 12;11:1431091. doi: 10.3389/fvets.2024.1431091 (PMC11670372; doi:10.3389/fvets.2024.1431091)
Supplement: Supplementary file 1 [file Table_1.docx]

**Supplementary Table 1:** Volume of electrolyte stock solution of digestion fluid at a concentration of 1.25X

|  | **SSF (pH 7)** | **SGF (pH 3)** | **SIF (pH 7)** |
| --- | --- | --- | --- |
| KCl (0.5 M) | 7.55 mL | 3.45 mL | 3.4 mL |
| KH_2_PO_4_ (0.5 M) | 1.85 mL | 0.45 mL | 0.4 mL |
| NaHCO_3_ (1 M) | 3.4 mL | 6.25 mL | 21.25 mL |
| NaCl (2 M) | - | 5.9 mL | 4.8 mL |
| MgCl_2_(H_2_O)_6_ (0.15 M) | 0.25 mL | 0.2 mL | 0.55 mL |
| (NH_4_)_2_CO_3_ (0.5 M) | 0.03 mL | 0.25 mL | - |
| HCl (6 M) | 0.045 mL | 0.65 mL | 0.35 mL |
| CaCl_2_(H_2_O_2_)_2_ (0.3 M) | 0.0125 mL | 0.0025 mL | 0.02 mL |
| H_2_O | 186.86 mL | 182.84 mL | 169.23 mL |
